# The effect of the number of hepatic inflow occlusion times on the prognosis of ruptured hepatocellular carcinoma patients after hepatectomy

**DOI:** 10.1186/s12893-022-01537-8

**Published:** 2022-03-13

**Authors:** Feng Xia, Zhiyuan Huang, Elijah Ndhlovu, Mingyu Zhang, Xiaoping Chen, Bixiang Zhang, Peng Zhu

**Affiliations:** 1 Center for Hepatic Surgery, Institute of HBP Surgery, Tongji Hospital of Tongji Medical College of Huazhong University of Science and Technology, Jiefang Avenue, Wuhan, 1095 Hubei China; 2 Department of Digestive Medicine Tongji Hospital of Tongji Medical College in Huazhong University of Science and Technology, Wuhan, Hubei China

**Keywords:** Ruptured hepatocellular carcinoma, Hepatic inflow occlusion, Hepatectomy

## Abstract

**Background and aim:**

It has been previously reported that inflow occlusion does not affect postoperative outcomes in hepatocellular carcinoma patients. However, for patients with ruptured hepatocellular carcinoma(rHCC), the effect of hepatic inflow occlusion and the number of occlusion times on the prognosis is unknown.

**Methods:**

203 patients with ruptured hepatocellular carcinoma were enrolled in this study. They were first divided into the non-hepatic inflow occlusion (non-HIO) group and the hepatic inflow occlusion (HIO) group. The Kaplan–Meier method was used to compare the recurrence-free survival and overall survival between the two groups. Patients in the HIO group were further divided into one-time HIO and two times HIO groups. KM method was also used to compare the two groups. Finally, independent risk factors affecting RFS and OS were determined by multivariate Cox regression analysis.

**Result:**

In the non-HIO group, 1-,3- and 5-year OS rates were 67.0%, 41.0%, and 22.0%respectively, and RFS rates were 45.0%, 31.0%, and 20.0% respectively; In the one-HIO group, the 1-,3-, and 5-year OS rates were 55.1%, 32.1%, and 19.2% respectively, and RFS rates were 33.3%, 16.7%, and 7.7% respectively; In the two-HIO group, 1-,3-, and 5-year OS rates were 24.0%, 0.0%, and 0.0% respectively, and RFS rates were 8.0%, 0.0%, and 0.0% respectively. By Cox regression analysis, HIO was an independent risk factor for a poor prognosis in rHCC patients.

**Conclusion:**

One time hepatic inflow occlusion did not affect postoperative OS, but negatively affected the RFS of rHCC patients; two times hepatic inflow occlusion negatively affected the postoperative OS and RFS in patients with rHCC.

## Introduction

Hepatocellular carcinoma (HCC) is one of the most serious cancers in the world and the second leading cause of death due to cancer. Rupture of hepatocellular carcinoma (HCC) is a rare and serious complication of HCC [1–5]. In recent years, the number of ruptured HCC(rHCC) patients has been increasing year by year, especially in Asia, and the proportion of rupture is much higher than in Europe and the United States. For patients with rHCC, transcatheter arterial chemoembolization (TACE), intraperitoneal chemotherapy, and hepatectomy have been used, but recent studies have shown that two-stage delayed hepatectomy is a relatively better treatment for suitable patients [6, 7]. Although rHCC patients have a good prognosis after hepatectomy, there is still a need to pay attention to their situation because ruptured HCC patients are prone to hemodynamic instability.

Since intraoperative bleeding is the main problem in hepatectomy, and intraoperative blood transfusion may affect the postoperative prognosis of patients, researchers have introduced the method of hepatic blood flow occlusion to control bleeding. At present, the most widely used techniques are the Pringle Maneuver and Hemihepatic inflow occlusion [8, 9]. The Pringle maneuver was first described in 1908 as a method that blocks hilar vessels and achieves the effect of controlling hepatic blood flow by clamping the hepatoduodenal ligament; in 1987, a hemihepatic occlusion (HHO) technique was proposed to control hepatic blood flow [10]. In short, both methods can effectively control hepatic blood flow, but they inevitably cause hepatic hypoperfusion and ischemia–reperfusion injury, and liver dysfunction occurs at the same time [11–13]. Patients with ruptured HCC are hemodynamically unstable on admission, and most of them have associated cirrhosis, reducing their tolerance to ischemia. Therefore, hepatic inflow occlusion can affect the liver function of the residual liver after surgery, and it may also affect the long-term prognosis of rHCC patients after surgery.

In the past, Jing-Hang Jiang et al. [14] believed that hepatic inflow occlusion did not affect the postoperative outcomes of HCC patients, while other researchers [15] found that HIO affected postoperative liver function, which in turn made the postoperative prognosis worse. However, the long-term effects of HIO on the prognosis of patients with ruptured HCC are unknown, and the effect of the number of times of HIO on prognosis is also unclear.

In this retrospective study, we aimed to assess the prognostic impact of HIO in patients with ruptured HCC. We also compared the effect of the number of HIO on the overall survival and recurrence-free survival of patients.

## Methods

### Patients

We retrospectively collected the data of 203 patients who were diagnosed with ruptured HCC and received surgical treatment at Wuhan Tongji Hospital between January 2010 and December 2018. We followed strict inclusion and exclusion criteria; the inclusion criteria were: (1) ruptured liver cancer determined by both contrast-enhanced CT and MRI (2) postoperative diagnosis of HCC confirmed by an experienced pathologist (3) liver function classification in Child–pugh class A or B (4) no invasion of the great vessels of the liver (5) negative resection margin (6) this admission was the first discovery of tumors; the exclusion criteria were: (1) postoperative diagnosis was not HCC (2) patients who had developed metastasis (3) patients who died within one month after surgery. Our research was authorized by the Ethics Committee of Tongji Hospital of Tongji Medical College of Huazhong University of Science and Technology (TJ-IRB20210205) [2021/02/04], and all patients gave informed consent.

### Propensity score matching analysis

Retrospective studies are prone to selection bias or confounding bias. Therefore, we used propensity score matching to reduce the selected bias. In this study, for patients undergoing hepatic inflow occlusion (HIO), there were differences in one variable. We included the BCLC stage in the propensity score model to balance the baseline. We performed 1:1 matching using SPSS 25.0. We chose a 0.1 caliper width so that an optimal trade-off can be obtained.

### Treatment mode

All patients included in the study were operated on by experienced surgeons at our liver surgery center. Whether hepatic inflow occlusion was to be performed was determined by the surgeon according to the intraoperative conditions, the extent of the tumor, and liver fibrosis or cirrhosis [16]. The Pringle Maneuver or hemihepatic vascular occlusion methods were chosen on a case-by-case basis in the HIO group. For these two methods, the time of each blocking was strictly limited to about 15 min, and the release interval was 5 min. Both HIO methods aim to reduce bleeding during parenchymal transection.

### Classification of postoperative complications

Because of the wide variety of complications and the relatively small number of patients with complications in each category, we used the Clavien-Dindo complication classification [17].

### Follow-up

All patients were followed up every 3 months within the first year and every six months after the second year after discharge, and all examinations included laboratory tests such as liver function, renal function, routine blood tests, tumor markers, and imaging tests such as enhanced abdominal CT and MRI. The time from the first day after operation to death or the last follow-up was defined as the overall survival (OS) rate, and the time from the first day after operation to the discovery of new lesions by physical and clinical examinations or the last follow-up was defined as the recurrence-free survival (RFS) rate. We set the time of the last follow-up to July 30, 2021.

### Data analysis

Continuous variables conforming to normal distribution are expressed by (mean ± standard deviation), and continuous variables not conforming to normal distribution are expressed by median (range). The differences between the two groups were compared using the independent samples t-test and Mann–Whitney U test, respectively, and the categorical data were analyzed using a fourfold table and a chi-square test. OS and RFS were calculated using the Kaplan–Meier method, and risk factors for OS and RFS were screened out by univariate and multivariate Cox regression. SPSS 25.0 statistical software and R (version 4.0.5, R Foundation for Statistical Computing, Vienna, Austria) were used for data processing.

## Results

### Basic characteristics of patients in the hepatic inflow occlusion (HIO) and non-HIO groups (before and after PSM)

A total of 203 patients with ruptured HCC were enrolled. The baseline data are shown in Table 1. We included gender, age, tumor length, tumor number, BCLC stage, Child–pugh classification of liver function, alpha-fetoprotein (AFP), Edmondson-Steiner classification, tumor necrosis, local tumor invasion, preoperative albumin (ALB), alanine aminotransferase (ALT), aspartate aminotransferase (AST), alkaline phosphatase (ALP), glutamyl transferase (GGT), HBsAg and other variables. Only the difference in the BCLC stage (P = 0.009) between the two groups was of statistical significance. The mean age of patients in the HIO group was 42.8 ± 11.4 years and 92.2% were male; the mean age of patients in the non-HIO group was 43.9 ± 11.7 years and 85.0% were male. Nearly 90% of all patients were HBsAg positive (Table 1). After 1:1 PSM correction, all variables in the non-HIO group were balanced, and the BCLC stage was not statistically different between the two groups (P = 1.000) (Table 2).

**Table 1:** Clinicopathological variables of ruptured HCC patients who underwent hepatectomy with hepatic inflow occlusion(HIO) and without hepatic inflow occlusion(non-HIO)

Variables	Non-hepatic inflow occlusion	Hepatic inflow occlusion	p-value
	n = 100	n = 103	
Gender (%)			0.104
Male	85 (85.0)	95 (92.2)	
Female	15 (15.0)	8 (7.8)	
Age (y)	43.9 ± 11.7	42.8 ± 11.4	0.498
Length (%)			0.437
≤ 5 cm	26 (26.0)	22 (21.4)	
> 5 cm	74 (74.0)	81 (78.6)	
Tumor number(%)			0.397
Single	81 (81.0)	78 (75.7)	
Multiple	19 (19.0)	25 (24.3)	
BCLC stage (%)			0.009
A	67 (67.0)	49 (47.6)	
B	20 (20.0)	25 (24.3)	
C	13 (13.0)	29 (28.2)	
Child–Pugh (%)			0.219
A	84 (84.0)	79 (76.7)	
B	16 (16.0)	24 (23.3)	
AFP (%)			0.470
≤ 400 ng/ml	39 (39.0)	35 (34.0)	
> 400 ng/ml	61 (61.0)	68 (66.0)	
Edmondson-steiner(%)			0.057
I	13 (13.0)	8 (7.8)	
II	36 (36.0)	54 (52.4)	
III	33 (33.0)	21 (20.4)	
IV	18 (18.0)	20 (19.4)	
Necrosis (%)			0.086
No	68 (68.0)	81 (78.6)	
Yes	32 (32.0)	22 (21.4)	
Local invasion (%)			0.437
No	46 (46.0)	53 (51.5)	
Yes	54 (54.0)	50 (48.5)	
ALB(g/L)	35.2 (32.3–38.6)	35.9 (33.1–38.8)	0.516
ALT(U/L)	27.0 (21.0–41.0)	28.0 (21.0–41.0)	0.548
AST(U/L)	36.5 (28.0–61.3)	37.0 (24.0–66.0)	0.919
ALP(U/L)	76.0 (56.0–93.0)	75.0 (61.0–93.0)	0.223
GGT(U/L)	47.0 (28.3–91.0)	58.0 (36.0–129.0)	0.074
HBsAg(%)			0.078
No	17 (17.0)	9 (8.7)	
Yes	83 (83.0)	94 (91.3)	

BCLC, Barcelona Clinic Liver Cancer; AFP, alpha fetoprotein; ALB, albumin; ALT, alanine aminotransferase; AST, aspartate aminotransferase; ALP, alkaline phosphatase; GGT, γ-glutamyl transpeptidase; HIO, hepatic inflow occlusion

**Table 2:** Clinicopathological variables of ruptured HCC patients who underwent hepatectomy with hepatic inflow occlusion(HIO) and without hepatic inflow occlusion(non-HIO) after PSM

Variables	Non-hepatic inflow occlusion	Hepatic inflow occlusion	p-value
	n = 80	n = 80	
Gender (%)			0.175
Male	65 (81.3)	72 (90.0)	
Female	15 (18.7)	8 (10.0)	
Age (y)	45.6 ± 11.4	44.4 ± 11.3	0.568
Length (%)			0.858
≤ 5 cm	21 (26.3)	22 (27.5)	
> 5 cm	59 (73.8)	58 (72.5)	
Tumor number (%)			0.855
Single	61 (76.3)	59 (73.8)	
Multiple	19 (23.8)	21 (26.3)	
BCLC stage (%)			1.000
A	48 (60.0)	48 (60.0)	
B	20 (25.0)	20 (25.0)	
C	12 (15.0)	12 (15.0)	
Child–Pugh (%)			0.454
A	64 (80.0)	59 (73.8)	
B	16 (20.0)	21 (26.3)	
AFP (%)			0.748
≤ 400 ng/ml	31 (38.8)	34 (42.5)	
> 400 ng/ml	49 (61.3)	46 (57.5)	
Edmondson-steiner (%)			0.091
I	11 (13.8)	7 (8.8)	
II	28 (35.0)	43 (53.8)	
III	26 (32.5)	16 (20.0)	
IV	15 (18.8)	14 (17.5)	
Necrosis (%)			0.205
No	56 (70.0)	63 (78.8)	
Yes	24 (30.0)	17 (21.3)	
Local invasion (%)			0.635
No	36 (45.0)	40 (50.0)	
Yes	44 (55.0)	40 (50.0)	
ALB (g/L)	35.3 (32.3–38.1)	35.6 (32.9–37.8)	0.889
ALT (U/L)	27.0 (21.0–43.3)	30.5 (24.0–44.0)	0.321
AST (U/L)	37.5 (28.5–63.5)	35.0 (22.3–65.8)	0.512
ALP (U/L)	76.0 (54.5–94.5)	76.5 (61.0–91.0)	0.475
GGT (U/L)	49.5 (28.0–93.3)	51.5 (35.0–126.8)	0.280
HBsAg (%)			0.159
No	14 (17.5)	7 (8.8)	
Yes	66 (82.5)	73 (91.3)	

BCLC, Barcelona Clinic Liver Cancer; AFP, alpha fetoprotein; ALB, albumin; ALT, alanine aminotransferase; AST, aspartate aminotransferase; ALP, alkaline phosphatase; GGT, γ-glutamyl transpeptidase; HIO, hepatic inflow occlusion; PSM, Propensity Score Matching

### Intraoperative and postoperative clinical results

In the non-HIO group, the average blood loss was 441 ± 183.5 ml, the average operation time was 321 ± 133.7 min, 41 patients had postoperative complications; in the HIO group, the average blood loss was 498.2 ± 258.3 ml, the average operation time was 305.4 ± 121.8 min, 36 patients had postoperative complications. There was no statistical difference in blood loss, operation time, and postoperative complications between the non-HIO group and the HIO group (Table 3).

**Table 3:** Comparison of intraoperative and postoperative findings

	Non-hepatic inflow occlusion	hepatic inflow occlusion	*P*
Blood loss (ml)			0.071
Mean(SD)	441.0(183.5)	498.2(258.3)	
Median (range[25%–75%])	282 (100–1200)	312 (80–1800)	
Duration of operation (min)			0.386
Mean(SD)	321.0(133.7)	305.4(121.8)	
Median (range[25%-75%])	279 (135–480)	298 (144–534)	
Dindo–Clavien morbidity			0.432
Grades I–IV	41	36	
I	16	17	
II	21	12	
III	3	6	
IIIa	3	5	
IIIb	0	1	
IV	0	1	
V	1	0	

SD, standard deviation

### Comparison of the OS and RFS in the HIO and non-HIO groups

In the HIO group, the median survival time was 358.0 days, the 1-, 3-, and 5-year OS rates were 47.6%, 24.2%, and 14.6% respectively; the 1-, 3-, and 5-year RFS rates were 27.2%, 12.6%, and 5.8% respectively; In the non-HIO group, the median survival time was 730 days, the 1-, 3-, and 5-year OS rates were 67.0%, 41.0%, and 22.0% respectively; the 1-, 3-, and 5-year RFS rates were 45.0%, 31.0%, and 20.0% respectively.

**Fig. 1:**
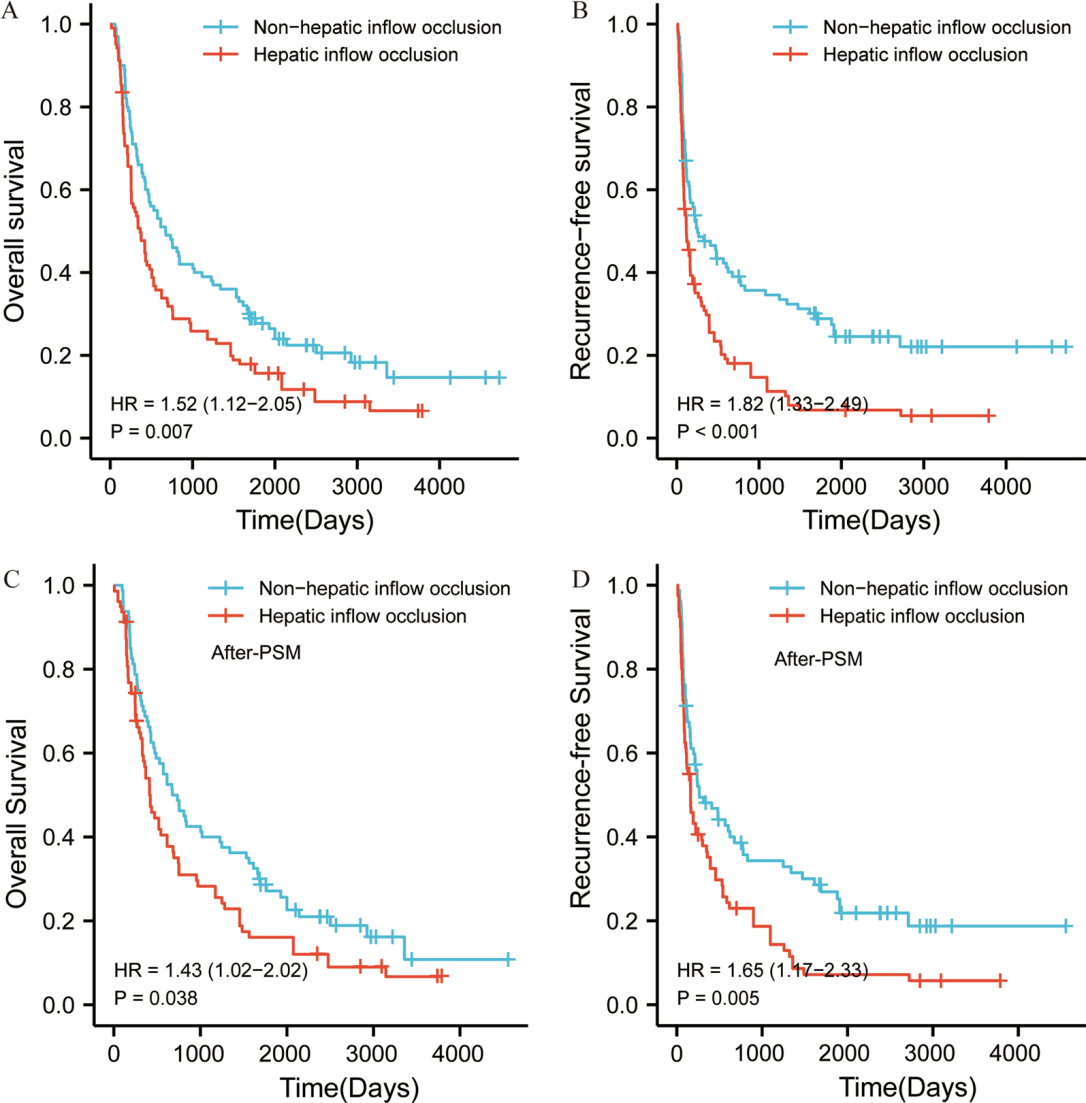
Overall survival and Recurrence-free survival curves of ruptured HCC patients in the HIO and non-HIO groups treated by hepatectomy. **A** represents OS (P = 0.007); **B** represents RFS (P < 0.001);** C** represents OS after PSM (P = 0.038);** D** represents RFS after PSM (P = 0.005).

Survival curves were plotted by the K-M method, and the OS (P = 0.007, HR = 1.52 (1.12–2.05)) and RFS (P = 0.001; HR = 1.82 (1.33–2.49)) were statistically different between the two groups (Fig. 1A and B). After PSM,survival curves were plotted by the K-M method, and the OS (P = 0.038, HR = 1.43 (1.02–2.02)) and RFS (P = 0.005; HR = 1.65 (1.17–2.33)) were also statistically different between the two groups (Fig. 1C and D).

### Basic characteristics of the patients in the one-time hepatic inflow occlusion group(one-HIO) and the two-times hepatic inflow occlusion group(two-HIO)

The HIO group was subdivided into the one-time HIO group (n = 78) and the two-times HIO group (n = 25. The included variables were the same as above. There was no statistical difference in all variables between the two groups (P > 0.05) (Table 4).

**Table 4:** Clinicopathological variables of ruptured HCC patients who underwent hepatectomy with one-time hepatic inflow occlusion(one-HIO) and two times hepatic inflow occlusion(two-HIO)

Variables	One time hepatic inflow occlusion	Two times hepatic inflow occlusion	p-value
	n = 78	n = 25	
Gender (%)			0.077
Male	74 (94.9)	21 (84.0)	
Female	4 (5.1)	4 (16.0)	
Age (y)	43.9 ± 11.4	39.1 ± 11.0	0.498
Length (%)			0.061
≤ 5 cm	20 (25.6)	2 (8.0)	
> 5 cm	58 (74.4)	23 (92.0)	
Tumor number (%)			0.567
Single	58 (74.4)	20 (80.0)	
Multiple	20 (25.6)	5 (20.0)	
BCLC stage (%)			0.2
A	41 (52.6)	8 (32.0)	
B	17 (21.8)	8 (32.0)	
C	20 (25.6)	9 (36.0)	
Child–Pugh (%)			0.654
A	59 (75.6)	20 (80.0)	
B	19 (24.4)	5 (20.0)	
AFP (%)			0.093
≤ 400 ng/ml	31 (39.7)	5 (20.0)	
> 400 ng/ml	47 (60.3)	21 (84.0)	
Edmondson-steiner (%)			0.115
I	7 (9.0)	1 (4.0)	
II	45 (57.7)	9 (36.0)	
III	14 (17.9)	7 (28.0)	
IV	12 (15.4)	8 (32.0)	
Necrosis (%)			0.136
No	64 (82.1)	17 (68.0)	
Yes	14 (17.9)	8 (32.0)	
Local invasion (%)			0.188
No	43 (55.1)	10 (40.0)	
Yes	35 (44.9)	15 (60.0)	
ALB (g/L)	35.6 (31.3–38.1)	36.5 (34.3–43.5)	0.053
ALT (U/L)	27.0 (20.5–41.8)	30.0 (24.0–49.0)	0.636
AST (U/L)	35.0 (22.0–65.3)	50.0 (31.5–80.0)	0.017
ALP (U/L)	77.0 (61.0–91.3)	72.0 (61.5–98.0)	0.661
GGT (U/L)	55.5 (36.0–122.3)	58.0 (29.5–147.5)	0.929
HBsAg (%)			0.881
No	7 (9.0)	2 (8.0)	
Yes	71 (91.0)	23 (92.0)	

BCLC, Barcelona Clinic Liver Cancer; AFP, alpha fetoprotein; ALB, albumin; ALT, alanine aminotransferase; AST, aspartate aminotransferase; ALP, alkaline phosphatase; GGT, γ-glutamyl transpeptidase; HIO, hepatic inflow occlusion

### Comparison of the OS and RFS in the one-HIO and two-HIO groups

In the one-HIO group, the median survival time was 469.3 days, the 1-, 3-, and 5-year OS rates were 55.1%, 32.1%, and 19.2% respectively; the 1-, 3-, and 5-year RFS rates were 33.3%, 16.7%, and 7.7% respectively; In the two-HIO group, the median survival time was 257.7 days,the 1-, 3-, and 5-year OS rates were 24.0%, 0.0%, and 0.0% respectively; the 1-, 3-, and 5-year RFS rates were 8.0%, 0.0%, and 0.0% respectively.

**Fig. 2:**
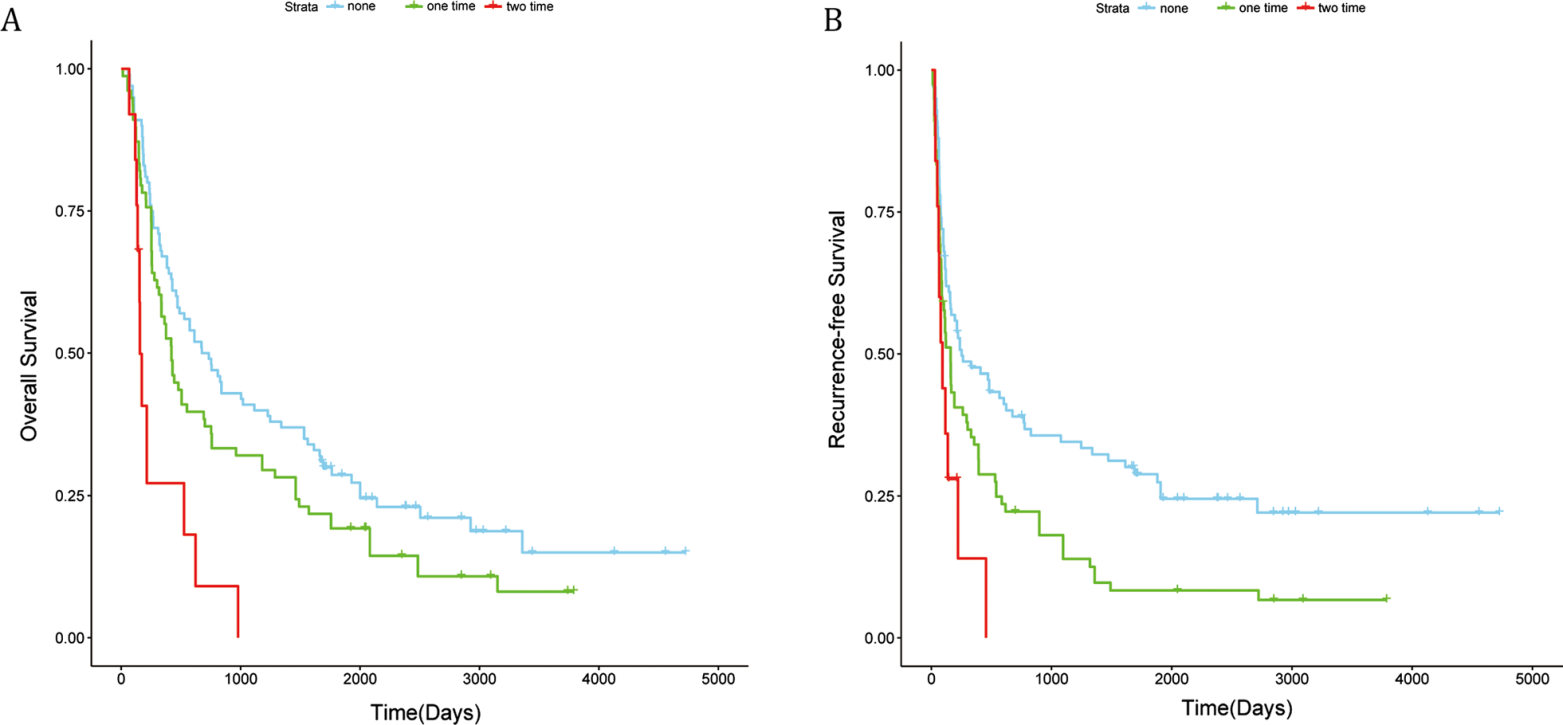
Overall survival and Recurrence-free survival curves of ruptured HCC patients in the one-HIO, two-HIO, and non-HIO groups treated by hepatectomy. **A** represents OS; **B** represents RFS

We plotted the survival curves using the K-M method, and the OS (P < 0.001, HR = 2.69 (1.63–4.44)) and RFS (P = 0.025, HR = 1.78 (1.07–2.96)) were statistically different between these two groups (Fig. 2).

### The one-HIO group and two-HIO group were compared with the non-HIO group for OS and RFS, respectively

According to the survival curve, when the one-HIO and the non-HIO group were compared, there was no statistical difference in OS (P = 0.088) between the two groups, and there was a difference in RFS (P = 0.003 HR = 1.63 (1.18–2.27)) between the two groups (Fig. 2); when the two-HIO and the non-HIO groups were compared, both the OS (P < 0.001, (HR = 3.64 (2.21–5.99)) and the RFS (P < 0.001 HR = 2.67 (1.61–4.43)) were statistically different between the two groups (Fig. 2).

### The impact of HIO on the prognosis of ruptured HCC patients was determined using Cox regression

To further determine the effect of HIO and the number of times of HIO on the postoperative prognosis of ruptured HCC patients, we used a multivariate Cox regression model to determine the risk factors affecting the postoperative OS and RFS of ruptured HCC patients.

In all 203 patients, we identified risk factors affecting OS and RFS by univariate and multivariate Cox regression models. In terms of the overall survival, HIO (P < 0.001) was a risk factor for decreased OS, but after stratifying for the number of occlusion times, one-HIO was not a risk factor for decreased OS (P = 0.495), and two-HIO was a risk factor for decreased OS (P < 0.001, HR = 4.116 (2.398–7.065)) (Table 5). For the RFS, not only was HIO (P < 0.001) a risk factor for decreased RFS, but both one-HIO((P = 0.003) HR = 1.643 (1.181–2.285)) and two-HIO ((P < 0.001) HR = 2.501 (1.521–4.112)) were also risk factors for decreased RFS (Table 6).

**Table 5:** Univariate and multivariate analysis to identify factors that predict overall survival in patients with ruptured hepatocellular carcinoma treated by hepatectomy

	Univariate analysis			Multivariate analysis		
	p	HR	95%confidence interval	p	HR	95%confidence interval
Gender						
Male/female	0.427	1.234	0.735–2.070			
Age						
Per year	0.720	1.003	0.987–1.019			
Tumor length						
Per cm	0.041	1.636	1.020–2.626	0.013	1.691	1.119–2.554
Tumor number						
Mutiple/single	0.021	1.822	1.095–3.034	0.016	1.862	1.120–3.096
BCLC	0.010			0.003		
B/A	0.621	0.873	0.509–1.496	0.822	0.941	0.552–1.603
C/A	0.002	2.000	1.296–3.087	0.003	1.880	1.239–2.852
Child–Pugh						
B/A	0.082	0.665	0.420–1.053			
AFP						
> 400 ng/ml/ ≤ 400 ng/ml	0.071	1.452	0.969–2.175			
Edmondson-steiner						
IV/III/II/I	0.637	1.046	0.969–2.175			
Necrosis						
Yes/no	0.485	0.872	0.867–1.263			
Local invasion						
Yes/no	0.002	1.718	0.592–1.282	0.001	1.686	1.223–2.323
ALB						
Per g	0.005	0.957	1.226–2.407	0.014	0.969	0.945–0.994
ALT						
Per U	0.007	0.990	0.927–0.987	< 0.001	0.987	0.980–0.994
AST						
Per U	0.010	1.006	0.982–0.997	0.001	1.007	1.003–1.011
ALP						
Per U	0.024	1.002	1.001–1.010	0.030	1.002	1.000–1.003
GGT						
Per U	0.775	1.000	0.998–1.002			
HBsAg						
Yes/no	0.242	0.736	0.440–1.230			
Times of HIO	< 0.001			< 0.001		
1/0	0.298	1.215	0.842–1.752	0.495	1.130	0.795–1.606
2/0	< 0.001	4.522	2.542–8.045	< 0.001	4.116	2.398–7.065
Blood loss						
> 400 ml/ ≤ 400 ml	0.023	1.322	1.182–1.744	0.144	1.288	0.892–1.286

BCLC, Barcelona Clinic Liver Cancer; AFP, alpha fetoprotein; ALB, albumin; ALT, alanine aminotransferase; AST, aspartate aminotransferase; ALP, alkaline phosphatase; GGT, γ-glutamyl transpeptidase; HIO, hepatic inflow occlusion

**Table 6:** Univariate and multivariate analysis to identify factors that predict recurrence-free survival in patients with ruptured hepatocellular carcinoma treated by hepatectomy

	Univariate analysis			Multivariate analysis		
	p	HR	95%confidence interval	p	HR	95%confidence interval
Gender						
Male/female	0.872	1.044	0.648–1.883			
Age						
Per year	0.470	0.994	0.983–1.013			
Tumor length						
Per cm	0.001	1.981	1.096–2.779	0.001	1.932	1.295–2.881
Tumor number						
Mutiple/single	0.171	1.460	0.783–2.370			
BCLC	0.118					
B/A	0.526	1.193	0.598–1.890			
C/A	0.039	1.594	0.927–2.254			
Child–Pugh						
B/A	0.169	0.717	0.450–1.163			
AFP						
> 400 ng/ml/ ≤ 400 ng/ml	0.112	1.312	0.812–1.823			
Edmondson-steiner						
IV/III/II/I	0.323	1.099	0.830–1.204			
Necrosis						
Yes/no	0.443	0.854	0.513–1.175			
Local invasion						
Yes/no	0.078	1.363	0.929–1.864			
ALB						
Per g	0.312	0.982	0.944–1.017			
ALT						
Per U	0.165	0.995	0.989–1.002			
AST						
Per U	0.224	1.002	0.998–1.006			
ALP						
Per U	0.965	1.000	0.998–1.002			
GGT						
Per U	0.001	1.004	1.002–1.006	< 0.001	1.003	1.002–1.005
HBsAg						
Yes/no	0.468	0.826	0.408–1.162			
Times of HIO	0.001			< 0.001		
1/0	0.007	1.659	1.158–2.405	0.003	1.643	1.181–2.285
2/0	0.001	2.604	1.505–4.491	< 0.001	2.501	1.521–4.112
Blood loss						
> 400 ml/ ≤ 400 ml	0.003	1.454	1.092–1.626	0.082	1.276	0.926–1.427

BCLC, Barcelona Clinic Liver Cancer; AFP, alpha fetoprotein; ALB, albumin; ALT, alanine aminotransferase; AST, aspartate aminotransferase; ALP, alkaline phosphatase; GGT, γ-glutamyl transpeptidase; HIO, hepatic inflow occlusion

In addition, tumor length,number of tumors, BCLC stage, local invasion, ALB, ALT, AST, and ALP were also independent risk factors affecting OS; and GGT and tumor length were also independent risk factors affecting RFS.

## Discussion

Rupture of liver cancer is a rare and serious complication of liver cancer with a high mortality rate. Although there are many treatment methods, such as TACE, intraperitoneal chemotherapy, surgical treatment, conservative treatment, etc., at present, the relatively better treatment is staged hepatectomy ( TACE for stage one treatment, followed by surgery as the second stage) [6]. Currently, there is an increasing number of research institutions discussing the risk factors affecting the postoperative survival of patients with ruptured HCC, hoping to find some prognostic factors to better manage rHCC patients [18–20]. In hepatectomy for rHCC patients, we need to pay attention to the amount of bleeding during surgery, because some rHCC patients are inherently hemodynamically unstable. Surgeons usually use hepatic inflow occlusion techniques, including the Pringle Maneuver and hemihepatic vascular occlusion to control the amount of bleeding. By blocking the blood flow into the liver, intraoperative blood loss is greatly reduced and the probability of blood transfusion is also reduced [8, 9, 21–23]. However, the adverse consequences of hepatic inflow occlusion should not be ignored. Both methods can cause postoperative liver function damage, and although some studies have indicated that HIO may not have an effect on the postoperative prognosis of HCC patients, the long-term survival rate of HIO in ruptured HCC patients is unknown, and the effect of the number of occlusion times on the prognosis is also unclear.

Previous studies have compared the Pringle Maneuver with hemihepatic blood flow occlusion, and there is still some controversy. In their meta-analysis, Wang et al. [23] found that the effects of the two techniques were not statistically different, but patients who were subjected to hemihepatic inflow occlusion had less liver injury. Similarly, Li et al. [24] and Chau et al. [15] found that hemihepatic inflow occlusion achieved similar results to the Pringle maneuver, but it had the advantage of reduced liver injury and better postoperative liver function recovery. While Yu et al. [25] concluded that the hemihepatic occlusion technique had the advantage of reduced operative time and blood loss, less injury, and better recovery when compared to the Pringle maneuver. In our study, the hepatic inflow occlusion method was selected according to the specific situation of the operator, and the blockage time was about 15 min each time. Although some studies indicated that hemihepatic blood flow blocking once could last for a longer time, considering that most patients had liver cirrhosis, the blockage time was set at about 15 min in our center. For patients with two blockages, the interval between each blockage was about 5 min.

Several previous retrospective studies [26–29] showed that there was no significant difference in postoperative long-term survival between HIO and non-HIO groups in HCC patients. Similarly, a meta-analysis [23] compared the effect of vascular occlusion in liver surgery on postoperative HCC patients, and the results showed that HIO did not affect the postoperative overall survival. In our study, we specifically studied the effect of HIO in ruptured HCC patients after surgery, while further analyzing the effect of the number of blockages on long-term survival. Our results showed that one HIO had no effect on postoperative OS but had a negative effect on RFS in patients with rHCC; Overall, HIO negatively affected both the postoperative OS and the RFS in patients with rHCC, which is different from previous studies on patients with HCC. Ischemia during hepatic portal blood flow occlusion is one of the factors that can negatively affect the overall survival, and ischemia–reperfusion injury(IRI) after occlusion may also harm liver function [30, 31]. However, only one occlusion did not affect the OS in our study, while two occlusions were associated with a reduction in the OS. So the total number of occlusions and the composite effect of multiple ischemia–reperfusion injuries may also differently affect the OS. The main mechanisms through which HIO affects recurrence can also be summed up in two points: (1) ischemia destroys the adhesion between tumor and endothelial cells, resulting in microvascular injury, and reperfusion injury promotes metastasis and growth of tumor cells [32–35]. (2) When in a blocked state, the pressure gradient between tumor vessels and portal vein can trigger cancer cells to detach from the main tumor, allowing tumor cells to translocate and spread.

Another point to note is why have most studies stated that HIO leads to tumor recurrence, while the conclusions for the postoperative OS of patients are very different? Lucinda Shen et al. [36] suggested that when performing HIO, they observed different blood flow responses of hepatic microvessels in different patients, which could also explain the different ischemia–reperfusion injury (IRI) effects on different patients when performing HIO, which in turn may lead to different postoperative OS’s. At the same time, it has been confirmed in some studies that changes in the circulation lead to heterogeneity in the response to IRI, and in livers with cirrhosis and fibrosis, the interaction between molecules disrupts the balance between cells and their surrounding matrix and also allows hepatic vascular remodeling. Many cells are involved in this process, including hepatic stellate cells, macrophages, and Kupffer cells. Hepatic endothelial cells are very important and help stabilize blood vessels. It is therefore necessary to develop techniques to perform intraoperative monitoring of hepatic microcirculation during hepatic inflow occlusions in the future [37].

Due to some selection or confounding bias, we also included all patient data in the multivariate Cox regression model, and the results showed that two times HIO was an independent risk factor affecting the postoperative OS of patients; HIO was an independent risk factor affecting the postoperative RFS of patients. In summary, our results suggest that HIO can affect the postoperative prognosis of ruptured HCC patients, but different blockage times will also affect the prognosis of patients differently.

This study has some limitations, namely: (1) This study is a retrospective study with some biases, and it is possible to perform a prospective study in the future to verify the conclusion. (2) The number of patients with ruptured HCC is relatively small, and the number of cases needs to be accumulated (3). Whether HIO affects postoperative complications has not been fully assessed, we need further investigation and follow-up.

In conclusion, HIO may affect the prognosis of patients with ruptured HCC, and the number of occlusion times can also affect the patients' prognosis. Although further RCTs are needed to validate this conclusion, in practical clinical work, we should consider the impact that HIO brings to patients with rHCC.

## Data Availability

The datasets used and analyzed in this study are available and can be obtained from the corresponding author on reasonable request.

## Abbreviations

BCLC: Barcelona Clinic Liver Cancer
HIO: Hepatic inflow occlusion (HIO)
HbsAg: Hepatitis B surface antigen
AFP: Alpha-fetoprotein
HCC: Hepatocellular carcinoma
rHCC: Ruptured hepatocellular carcinoma
CT: Computed tomography
MRI: Magnetic resonance imaging
RFA: Radiofrequency ablation
TACE: Transcatheter arterial chemoembolization
OS: Overall survival
RFS: Recurrence-free survival
MVI: Microvascular invasion
ALB: Albumin
ALT: Alanine aminotransferase
AST: Aspartate Transaminase
ALP: Alkaline phosphatase.
GGT: Glutamyl transpeptidase
